# It’s Not Easy Being Blue: Are There Olfactory and Visual Trade-Offs in Plant Signalling?

**DOI:** 10.1371/journal.pone.0131725

**Published:** 2015-06-26

**Authors:** Kim Valenta, Kevin A. Brown, Amanda D. Melin, Spencer K. Monckton, Sarah A. Styler, Derek A. Jackson, Colin A. Chapman

**Affiliations:** 1 Department of Anthropology, McGill University, Montreal, Quebec, Canada; 2 Dalla Lana School of Public Health, University of Toronto, Toronto, Ontario, Canada; 3 Department of Anthropology, Campus Box 1114, One Brooking Drive, Washington University, St. Louis, Missouri, United States of America; 4 Department of Biology, York University, Toronto, Ontario, Canada; 5 Leibniz Institute for Tropospheric Research, Leipzig, Germany; 6 Department of Chemistry, University of Toronto, Toronto, Ontario, Canada; Instituto de Biología Molecular y Celular de Plantas (IBMCP), SPAIN

## Abstract

Understanding the signals used by plants to attract seed disperses is a pervasive quest in evolutionary and sensory biology. Fruit size, colour, and odour variation have long been discussed in the controversial context of dispersal syndromes targeting olfactory-oriented versus visually-oriented foragers. Trade-offs in signal investment could impose important physiological constraints on plants, yet have been largely ignored. Here, we measure the reflectance and volatile organic compounds of a community of Malagasy plants and our results indicate that extant plant signals may represent a trade-off between olfactory and chromatic signals. Blue pigments are the most visually-effective – blue is a colour that is visually salient to all known seed dispersing animals within the study system. Additionally, plants with blue-reflecting fruits are less odiferous than plants that reflect primarily in other regions of the colour spectrum.

## Introduction

The physical properties of fruits can act as signals of edibility and nutrition to the seed dispersing animals that play a crucial role in moving seeds away from the parent tree and, thus, increasing plant fitness [1,2,3,4,5]. Numerous studies have demonstrated that plant signals and cues are critical to fruit selection by animals [6,7,8,9]. While ripe fruit signals refer to traits such as colour and odour that are maintained by natural selection because of their ability to reliably convey information to other organisms [10], ripe fruit cues refer to traits that evolved in a context unrelated to animal signalling that may nonetheless convey reliable information to dispersers [11]. Plant signals and cues take a multitude of forms, including fruit chromaticity, odour, and size [7,12,13,14], and have been shown to reliably advertise fruit nutrient content to dispersers [15]. Given variation in disperser sensory abilities, including colour vision and olfactory ability, fruit signals and cues may result in trade-offs between fruit colour and odour signals.

The fruit syndrome hypothesis posits that suites of fruit signals should be directed at the frugivorous guilds that provide the highest quality seed dispersal service, according to their capacity to receive signals [14]. The fruit traits of a given species are, at least in part, predicted to be the subset of the fruit phenotype spectrum reflecting the selective pressures exerted by beneficial seed dispersers [16,17]. One of the most compelling dichotomies in such selective pressures is the conflict between olfactory and chromatic signals. Plants can signal fruit presence and ripeness through visual and olfactory channels [18]. In systems where beneficial dispersers rely variously on visual and olfactory signals, there may be a selective tension imposed on plants. Plants may produce olfactory signals to increase detection by olfactory-driven foragers (e.g., nocturnal and dichromatic mammals), or visual signals to attract visually-oriented foragers (e.g., diurnal avian frugivores). In systems with mixed animal disperser assemblages that include both olfactory and visually oriented frugivores, plant signals may thus represent a tradeoff between fruit colour and odour.

The fruit syndrome hypothesis has intuitive appeal and support for the colour-odour trade-off has been shown to exist for bird- versus bat-dispersed species [14] and in multiple taxon-specific studies [19,20,21]. However, this topic is heavily debated because most fruits are dispersed by multiple taxa possessing diverse sensory phenotypes [16,22,23]. As such, specialized fruit traits targeting a restricted set of seed dispersers may be selected against [4] or result in limited fruit diversification, and therefore be limited to relatively few plant taxa [24,25]. Finally, convergence of fruit traits among different phylogenetically diverse plant species dispersed by different frugivorous guilds has been argued to refute the hypothesis that specific frugivore species are driving the evolution of fruit morphology and generating syndromes [16,26]. Alternately, fruit trait convergence may result from phylogenetic constraint [27].

Our study aims to help clarify this debate by approaching the fruit syndrome hypothesis using a quantitative and multivariate approach. For the fruit syndrome hypothesis to be supported, there should be a negative relationship between chromatically conspicuous fruits and odiferous fruits, i.e., fruits that invest in colour should not invest in odour, due to the predicted trade-off between attracting olfactory-driven versus visually oriented foragers.

Here, we evaluate the fruit syndrome hypothesis by testing whether fruits invest in specific signalling strategies at the expense of others. To evaluate potential phylogenetic constraint on these strategies, we first test for the presence of a phylogenetic structure among traits using a species-level phylogeny. We examine chromatic (visual) and odour (olfactory) signals in an analysis of 56 endemic wild fruit species in a tropical dry forest in Madagascar. We predict that fruits that invest in pigment production in non-photosynthetically active regions of the chromatic spectrum will not invest heavily in odour production. Additionally, because plants with a higher surface-area-to-weight ratio (i.e., smaller fruits) produce more signals relative to overall fruit production (i.e. while the magnitude of signal production may be similar to that produced by larger fruits, the amount of signal relative to the amount of fruit produced will be greater) we predict that the relationship between fruit colour and odour will be altered by surface-area-to-weight ratio of fruits.

## Materials and Methods

### Colour and odour quantification

Ethics statement—N/A. Permission for fieldwork granted by Madagascar National Parks and the Government of Madagascar (permit number: 092N_EA07/MG12). Between January and December, 2012, between 5–10 ripe fruits of each species were opportunistically collected in the Ampijoroa region of Ankarafantsika National Park, Madagascar (15' '59' -16°22S, 47''56'-47''12E). Ripe fruits of each species were collected directly from trees and all analyses were performed within ~2 hours of fruit collection. Fruit ripeness was determined based on colour, odour, and hardness and confirmed after analysis based on the presence of viable seeds. Seeds were considered viable if they were fully formed, had the approximate mass of seeds known to germinate, and had no evidence of damage. Plants were identified to genus and species using a published tree flora [28] and an unpublished photographic database of the plants of the national park (Sato, pers comm). All known genera were assigned to family using a published tree flora [28] and assigned an order based on the APG III classification [29]. In total, the analysis included species belonging to 19 families and 10 orders (Table 1). In cases where it was not possible to identify plants to the genus level, they were identified either by their local Malagasy name, or categorized as unknown, each of which was given a unique number. Ripe fruits were weighed using a digital scale and measured in three dimensions using calipers.

**Table 1:** Fruit traits of sampled species including surface-area-scaled VOC sum, percent reflectances per band normalized by brightness, wavelength of peak reflectance in nm (λmax; peak hue), peak reflectance value (brightness), surface area:weight ratios, and number of fruits sampled.

Species	Family	Order	VOC	UV Reflectance	Blue Reflectance	Green Reflectance	Red Reflectance	λmax	Peak Brightness	Area to Weight	N Fruits
*Androstachys spp*	Euphorbiaceae	Malpighiales	1.16	4.14	24.12	37.66	38.21	655	0.32	318.48	10
*Antidesma petiolare*	Euphorbiaceae	Malpighiales	34.34	1.99	13.01	46.27	40.72	700	0.34	849.49	5
*Asterotrichilia asterotricha*	Meliaceae	Sapindales	1.92	12.39	17.99	51.87	30.15	557	0.18	229.67	5
*Asterotrichilia spp*	Meliaceae	Sapindales	3.97	18.81	18.05	57.11	24.83	565	0.23	173.84	5
*Asterotrichilia marina*	Meliaceae	Sapindales	1.78	29.71	25.09	47.06	27.85	553	0.33	279.42	5
*Badouinia fluggeiformis*	Fabaceae	Fabales	5.22	8.7	11.63	59.83	28.54	700	0.4	373.06	5
*Baudouinia spp*	Fabaceae	Fabales	5.26	8.7	11.63	59.83	28.54	655	0.33	548.61	5
*Berchemia discolor*	Rhamnaceae	Rosales	4.51	0.19	0	95.86	4.14	562	0.24	350.79	5
*Bridellia pervilleana*	Euphorbiaceae	Malpighiales	5.86	2.75	1.95	59.98	38.07	691	0.09	662.4	10
*Croton spp*	Euphorbiaceae	Malpighiales	7.58	11.76	6.34	62.38	31.27	668	0.06	1291.5	10
*Croton spp 2*	Euphorbiaceae	Malpighiales	4.98	7.59	11.85	35.71	52.44	684	0.43	1340.7	10
*Elaeocarpus subserratus*	Elaeocarpaceae	Malvales	38.8	20.1	18.36	48.64	33	698	0.41	532.14	5
*Empogona ovalifolia*	Rubiaceae	Gentianales	10.3	15.58	11.02	60.39	28.59	700	0.21	720.51	5
*Gaertnera spp*	Rubiaceae	Gentianales	2.75	3.98	2.13	64.58	33.28	561	0.12	630.8	10
*Garcinia arenicola*	Clusiaceae	Malpighiales	1.28	7.89	8.37	56.41	35.23	698	0.12	255	5
*Gardenia rutenbergiana*	Rubiaceae	Gentianales	5.34	10.52	11.11	59.58	29.31	700	0.18	181.83	5
*Grangeria spp*	Chyrsobalanaceae	Rosales	9	3.79	9.41	45.44	45.15	699	0.34	429.01	5
*Grewia madagascariensis*	Malvaceae	Malvales	13.74	1.66	8.98	37.81	53.2	700	0.62	490.3	5
*Grewia triflora*	Malvaceae	Malvales	24.18	7.55	7.42	50.27	42.32	564	0.14	638.07	10
*Landolphia myrtifolia*	Apocynaceae	Gentianales	0.22	5.4	10.6	60.31	29.09	700	0.34	113.22	5
*Mapouria boinensis*	Rubiaceae	Gentianales	10.95	7.62	5.35	47.46	47.2	699	0.32	478.6	8
*Mapouria spp*	Rubiaceae	Gentianales	15.86	5.96	7.63	54.72	37.65	700	0.51	469.4	10
*Monanthotaxis valida*	Annonaceae	Magnoliales	10.93	5.5	9.08	56.44	34.48	700	0.6	410.89	5
*Noronhia spp*	Oleaceae	Lamiales	38.91	6.51	12.96	57.33	29.72	700	0.36	466.56	5
*Petchia spp*	Apocynaceae	Gentianales	1.79	20.67	26.28	35.2	38.52	669	0.84	1964.4	5
*Rothmania renniformis*	Rubiaceae	Gentianales	2.56	15.49	13.02	57.05	29.93	646	0.11	242.75	5
*Rourea orientalis*	Connaraceae	Rosales	4.22	15.46	17.66	52.64	29.7	692	0.49	506.1	5
*Salvadora augustifolia*	Salvadoraceae	Brassicales	1.98	13.44	15.15	44.4	40.44	563	0.31	758.21	5
*Sorindeia madagascariensis*	Anacardiaceae	Sapindales	1.39	11.59	19.83	37.5	42.67	631	0.21	244.17	6
*Strychnos decussata*	Loganiaceae	Gentianales	3.08	8.55	11.62	56.03	32.35	700	0.44	247.57	5
*Strychnos madagascariensis*	Loganiaceae	Gentianales	3.84	7.25	9.85	64.61	25.54	700	0.46	153.01	6
*Strychnos spp*.	Loganiaceae	Gentianales	4.21	2.66	6.04	65.22	28.75	697	0.62	240.62	5
*Strychnos myrtoides*	Loganiaceae	Gentianales	4.16	7.09	13.82	57.22	28.96	661	0.19	493.08	5
*Strychnos spinosa*	Loganiaceae	Gentianales	0.25	4.93	10.59	46.77	42.65	699	0.48	66.61	5
*Tabernaemontana coffeeoides*	Apocynaceae	Gentianales	2.52	5.43	27.41	35.49	37.1	571	0.43	325.88	5
*Terminalia trophophylla*	Combretaceae	Myrtales	23.3	7.44	8.65	59.68	31.67	551	0.17	1047.7	5
*Tina isaloensis*	Sapindaceae	Sapindales	2.26	58.39	32.85	28.59	38.56	692	0.3	1200.6	5
*Tricalysia perrieri*	Rubiaceae	Gentianales	15.75	12.92	13.03	59	27.96	697	0.21	614.93	10
*UK Species 1*	UK	UK	4.59	14.74	15.7	56.12	28.19	678	0.1	547.35	10
*UK Species 2*	UK	UK	13.95	16.85	13.47	51.04	35.49	638	0.21	157.38	5
*UK Species 3*	UK	UK	6.45	31.96	24.97	34.21	40.82	679	0.5	470.76	5
*UK Species 4*	UK	UK	0.5	12.02	11.76	58.25	29.99	697	0.39	123.83	5
*UK Species 5*	UK	UK	9.73	5.96	1.11	66.82	32.08	568	0.29	884.36	10
*UK Species 6*	UK	UK	23.25	23.04	4.85	71.76	23.39	567	0.17	674.54	10
*UK Species 7*	UK	UK	5.76	18.87	15.16	51.35	33.49	679	0.51	567.53	5
*UK Species 8*	UK	UK	45.91	3.42	8.11	59.15	32.75	580	0.19	428.23	5
*UK Liana 1*	UK	UK	9.35	2.9	3.93	56.83	39.24	578	0.12	530.81	5
*UK Liana 2*	UK	UK	1.94	5.19	4.47	58.64	36.89	562	0.24	316.26	5
*UK Liana 3*	UK	UK	2.81	15.61	26.17	38.72	35.11	699	0.18	582.08	10
*UK Liana 4*	UK	UK	17.14	22.94	4.03	45.37	50.6	700	0.21	447.56	6
*UK Liana 5*	UK	UK	53.44	12.79	3.44	65.51	31.05	646	0.27	513.3	5
*Vitex beraviensis*	Lamiaceae	Lamiales	1.08	11.78	11.81	57.02	31.17	684	0.1	106.74	5
*Vitex perrieri*	Lamiaceae	Lamiales	4.83	6.5	13.98	45.2	40.83	646	0.27	436.45	5
*Vitex spp*	Lamiaceae	Lamiales	1.28	3.89	9.06	54.64	36.3	630	0.13	311.88	5
*Vitex spp 1*	Lamiaceae	Lamiales	4.56	6.5	13.98	45.2	40.83	693	0.1	426.36	5
*Ximenia caffra*	Oleaceae	Lamiales	1.54	5.4	14.54	47.23	38.23	699	0.08	491.01	5

Peak reflectance values are the proportion reflectance across wavelengths of light in 1-nm increments (400-700nm). Brightness values have been normalized such that the lowest value in the 400-700nm range is set to zero to account for potential spectrometer drift across measurements.

To quantify fruit odour, we measured volatile organic compound (VOC) emissions of ripe fruits. Ripe fruits were placed in inert ~1.5 L plastic sampling bags and the atmosphere within each bag was sampled using a vacuum pump (Gilian 5000, Sensidyne) that pulled air through the sample bag (1L/min, 240 minutes) and into two odourant-adsorbent filters (Amberlite XAD-2, 400-200mg, Sigma-Aldrich). Contamination of the sampling enclosure with ambient VOCs was minimized by passing incoming air through a container of activated carbon. Additionally, blank samples were collected and analysed to identify contamination from the sampling apparatus. Three peaks representing sampling apparatus contamination were detected in each blank sample and these peaks were subtracted from total VOC sums of fruit samples. Trapped VOCs were analysed using the procedure and instrumentation reported in [19]. All VOC sums were divided by the surface area of sampled fruits to obtain VOCs per unit surface area.

**Fig 1:**
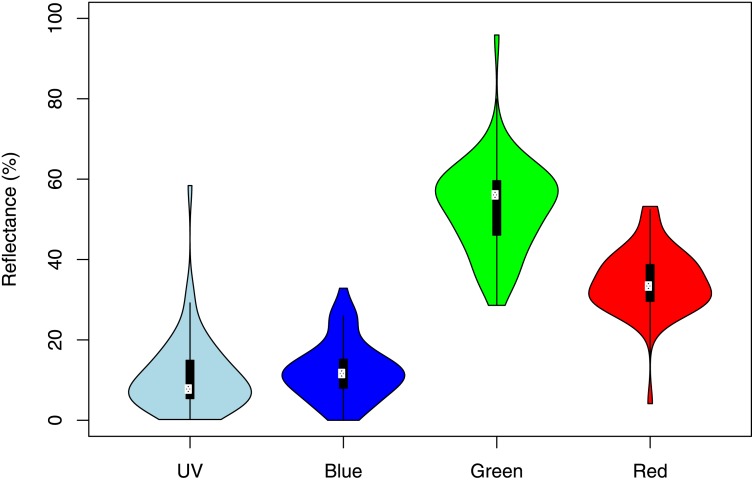
Univariate violin plots showing the reflectance for all fruits in each of the four colour reflectance bands. Ultraviolet (300-400nm), blue (400-500nm), green (500-600nm), and red (600–700 nm). For each reflectance band, the white dot corresponds to the median, while the lower and upper end of the thick black bars correspond to the 25^th^ and 75^th^ percentiles. The width of the violin plot represents the density of the distribution.

**Fig 2:**
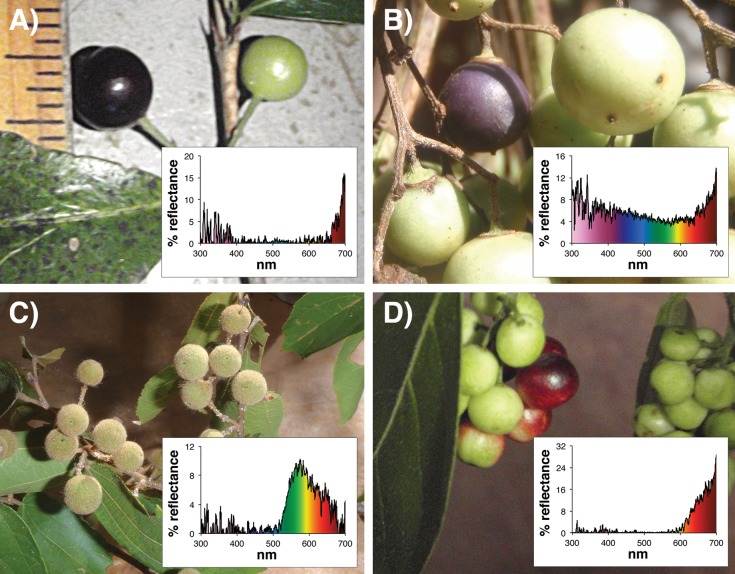
Photographs and associated spectrograms showing four fruits, and their associated reflectance spectra. **A**) *Tricalysia perrieri*, **B**) UK Liana 3, **C**) *Grewia triflora*, **D**) *Antidesma petiolare*. Photo credit: KV.

Reflectance spectra of one ripe fruit of each species were measured relative to a Spectralon white reflectance standard (Labsphere) on-site using a Jaz portable spectrometer and a PX-2 pulsed xenon lamp (Ocean Optics Inc.) emitting a D-65 light source, with a range of 250-720nm (Figs 1 and 2). The fruit scanning angle was fixed at 45° and external light was blocked using thick black fabric.

### Colour and Odour Measures

We used a VOC index, calculated as log_10_ surface-area-scaled sum of VOC emissions. We calculated the surface area of each fruit using the following equation for the surface area of an ellipsoid: 4π[((ab)^p^+(bc)^p^+(ac)^p^)/3] ^1/p^ where p = 1.6075 [19]. Four reflectance indices were calculated; ultraviolet (UV, 300-400nm), blue/violet (400-500nm), green/yellow (500-600nm), and orange/red (600-700nm) wavelengths. To control for brightness, the four reflectance indices were calculated as the reflectance in the specified 100nm band divided by the sum of reflectance in the visible range (400-700nm). Brightness was standardized in the visible range because reflectance in the visible range comes at the cost of photosynthetic absorption. Conversely, absorption in the ultraviolet range can result in photoinhibition—absorption of light in this spectrum can be damaging to plants [30]. Reflectance in the ultraviolet range is therefore beneficial in terms of avoiding photoinhibition, and additionally it does not come at a photosynthetic absorption cost. Thus, we chose this method because we wanted to compare relative reflectance within the visible light range (e.g., proportion of blueness, versus redness). This method allows us to compare the relative reflectance of light in the region where it is potentially photosynthetically costly to reflect light (400-700nm). For more detailed multivariate analyses, individual reflectance values (n = 1137) across the 300-700nm range were used directly after normalizing them by the total reflectance in the 400-700nm range, as above.

### Phylogenetic Methods

VOC emission, UV, blue, green, and red reflectance were optimized onto a species-level phylogeny as continuous characters, using TNT version 1.1 [31]. The framework phylogeny was adapted from APG III and other classifications [29,32,33,34,35,36] (S1 Fig). The above characters were mapped onto the model phylogeny for a maximally parsimonious arrangement, such that a range of values was optimally assigned to each node. If any of these traits had a higher-level phylogenetic basis we would expect similar values to cluster within a taxon. No such patterns were observed, with optimal character distributions having extensive homoplasy. Assuming maximum parsimony, one would expect a phylogenetically informative trait to exhibit minimal homoplasy, so we calculated consistency and retention indices for each trait (CI and RI, respectively). CI is a direct estimate of homoplasy (i.e., from 0 to 1, CI = 1 if there is none), while RI approximates how well the phylogenetic tree fits a character (i.e., from 0 to 1, RI = 1 if fit is perfect); thus, if any of the measured traits are phylogenetically informative, both values should fall closer to 1 [37]. For a formal test of phylogenetic signal, Blomberg’s K [38] and Pagel’s λ [39] were calculated in R (R Core Development Team, 2014) using the phytools package [40] and compared to a null model using the likelihood-ratio test (S1 File).

### Statistical Methods

To assess the association between fruit odour production and fruit chromaticity, we calculated the Pearson correlation of UV, blue, green, and red reflectance with VOC index. To account for correlation across normalized spectrum-reflectance values, we developed a multivariate regression model that included a spline transformation of the spectrum-reflectance values. A series of five predictor variables were calculated as weighted sums of spectrum-reflectance values. The weights for the five predictor variables were determined by a natural-cubic spline that had knots at 350, 410, 470, 530 and 590 nm. Because the summed reflectance values between 400 and 700 nm added to 100%, our spline transformation used the 650nm bandwidth as a fixed referent value to protect against multi-collinearity [41]. Performing the regression using this series of variables rather than the UV, blue, green and red reflectance variables enabled us to model a smooth association between spectrum and VOC. Using the spline-transformed spectrum-reflectance as the principal explanatory variables, we ran a multivariate model that included the log transformed fruit surface-area-to-weight ratios. Because linear regression models handle multiple independent variables but only a single dependent variable, the transformed spectrum-reflectance values were considered as the independent variables in the model. We used this analysis to estimate an adjusted association—an association between variables while holding all other variables constant—and not to imply that fruit colour differences cause changes in VOC production (and not the inverse). For each 100nm colour band, we back-calculated the cumulative coefficient and the variance using the delta method [41]. All analyses were calculated in R (R Core Development Team, 2014), and reported p-values are based on two-tailed hypothesis testing (S2 File).

## Results and Discussion

When mapped onto a model phylogeny, none of the measured fruit traits showed any evidence of being phylogenetically informative. Calculated values of CI (maximum = 0.291, UV reflectance) and RI (maximum = 0.386, red reflectance) were much lower than would be sufficient for an informative character [37]. We detected no significant phylogenetic structure for any trait using Blomberg’s K and Pagel’s λ: VOC (K = 0.28, p = 0.59; λ<0.001, p = 1.0), UV (K = 0.36, p = 0.16; λ = 0.22, p = 0.40), blue (K = 0.42, p = 0.23; λ = 0.54, p = 0.24), green (K = 0.36, p = 0.30; λ = 0.28, p = 1.0), red (K = 0.37, p = 0.26; λ = 0.72, p = 0.42) (Table 2). We therefore conclude that traits are not phylogenetically constrained.

**Table 2:** Results from analyses of fruit traits using a model species-level phylogeny.

	CI	RI	K	p	Λ	p
VOC	0.180	0.173	0.28	0.59	0.00	1.00
UV	0.291	0.275	0.36	0.16	0.22	0.40
Blue	0.193	0.270	0.42	0.23	0.54	0.24
Green	0.230	0.290	0.36	0.30	0.28	1.00
Red	0.260	0.386	0.37	0.26	0.72	0.42

P-values for Blomberg’s K and Pagel’s λ are from likelihood-ratio tests.

**Fig 3:**
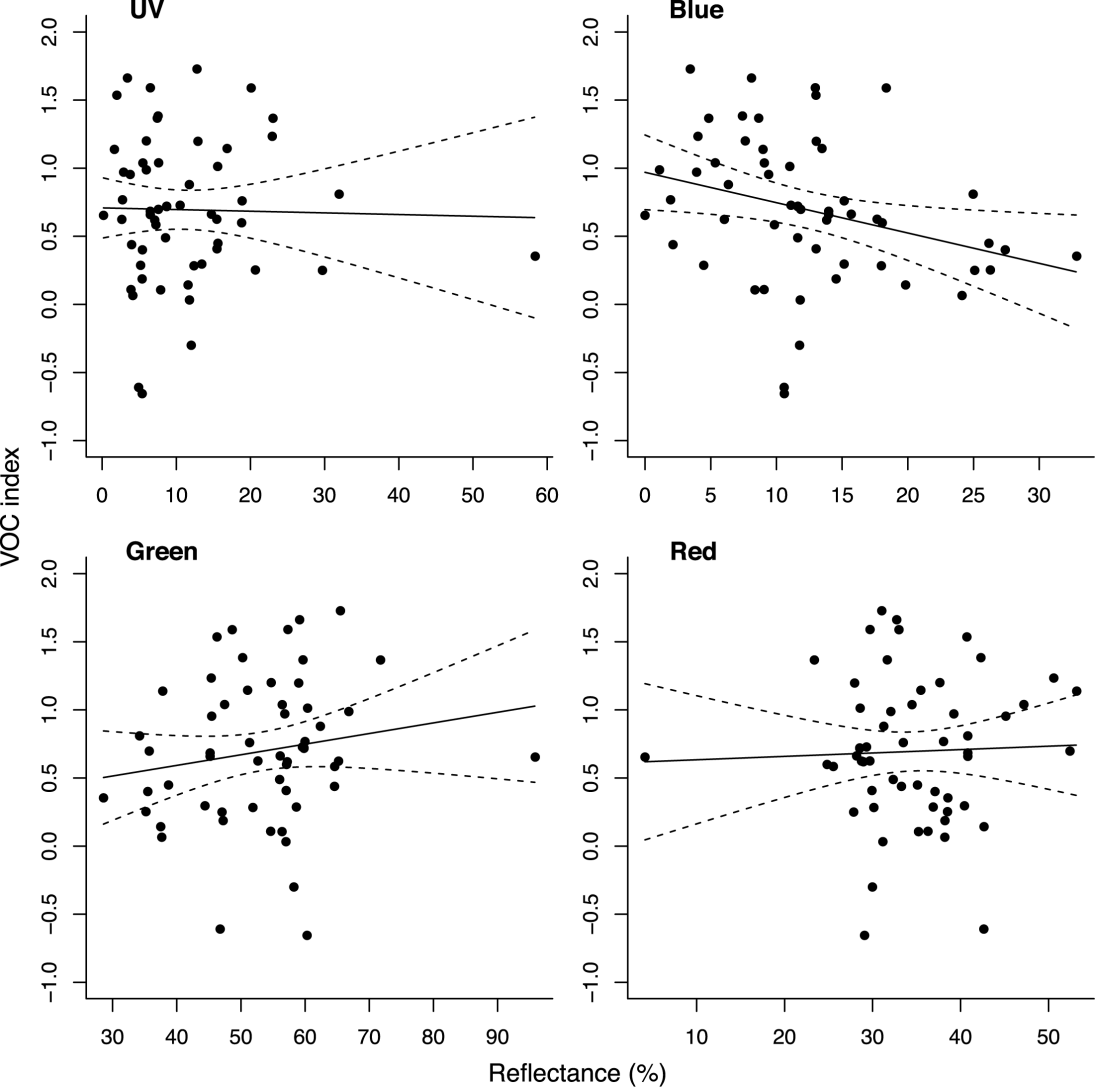
Bivariate scatter plots showing the relationship between overall odour emission (VOC) and reflectance in each of the four colour reflectance bands. UV (300-400nm), blue (400-500nm), green (500-600nm), and red (600-700nm). Percent reflectance was calculated as regions of the spectrum reflecting in the specified 100nm band divided by the sum of reflectance in the visible range (400-700nm).

There was a significant negative relationship between blue reflectance and overall VOC index (r = -0.32, p = 0.02). We observed no significant relationship between VOC index and any of the other reflectance ranges: UV (r = -0.01, p = 0.94), green (r = 0.14, p = 0.32), or red reflectance (r = 0.04, p = 0.76; Fig 3). The surface-area-to-weight ratio was a highly significant predictor of the VOC index. Smaller fruits with higher surface-area-to-weight ratios had substantially higher VOC emissions (r = 0.50, p < 0.001) (Table 3).

**Table 3:** Pearson correlation coefficients for fruit traits of sampled species (N = 56) including log transformed surface-area-scaled VOC sum, percent reflectances per band normalized by brightness, surface area:weight ratios.

	VOC	UV Reflectance	Blue Reflectance	Green Reflectance	Red Reflectance	Surface Area to Weight Ratio
VOC						
UV Reflectance	-0.01					
Blue Reflectance	-0.32*	0.43**				
Green Reflectance	0.14	-0.55**	-0.74**			
Red Reflectance	0.04	-0.33*	0.00	-0.49**		
Surface Area to Weight Ratio	0.50**	0.17	-0.01	-0.18	0.14	

* p < 0.05

** p < 0.01

**Fig 4:**
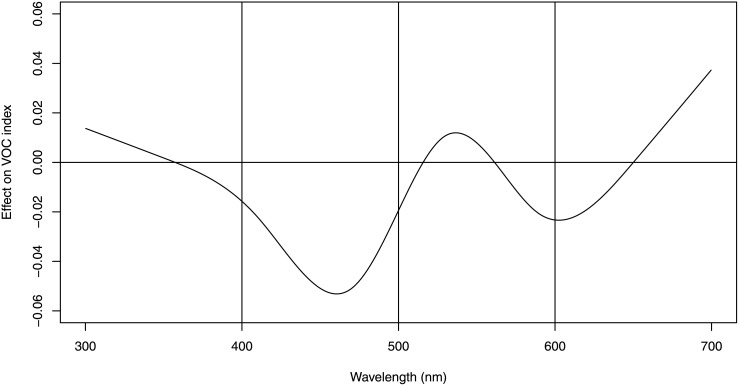
The relationship between overall odour emission (log_10_ VOC) and reflectance across the 300nm to 700nm colour range. This analysis was based on a natural cubic spline transformation of the spectrum values (300-700nm). Each one-unit increase of reflectance in the blue spectrum (400-500nm) was associated with an 11% decrease (log_10_ effect = -0.039, p = 0.009) in VOC while reflectance in the UV, green and red spectra were not associated with VOC.

To control for the confounding effect of fruit size and correlated reflectance bands, we ran a multivariate model that included a spline transformation of the normalized reflectance values (Fig 4). The figure demonstrates that higher reflectance in the 400nm to 600nm range was associated with lower VOC in the fruit sample. Overall, reflectance was a significant predictor of VOC emissions (5 d.f., F = 2.8, p = 0.03). As with our bivariate analyses, we found a substantial negative association between VOC and blue reflectance (log_10_ effect = -0.039, p = 0.009, R^2^ = 0.41) and a lack of relationship in the other reflectance bands.

Our prediction that fruit investment in non-photosynthetically active pigments would scale negatively with odour production is partially supported. We find that bluish fruits have significantly lower total VOC emissions than fruits reflecting other hues, which is consistent with the fruit syndrome hypothesis. Specifically our results suggest the existence of a disperser-mediated dichotomy in fruit colour and odour signal production: plants that invest more in blue chromatic signals invest less in odour, yet fruit size is a critical component.

The trade-off between blue chromatic reflectance and VOC emission is particularly compelling because reflectance in the blue range of the spectrum (400-500nm) may be doubly costly to a plant as it occupies critical photosynthetic space—both chlorophyll A and B absorb blue wavelengths of light [42]. Thus plants investing in pigments (e.g., indigoids, anthocyanins) that reflect light at this range of the spectrum may be investing in both pigment production with a concomitant loss of photosynthetic potential [43]. Alternatively, chromatic reflectance and VOC emissions could reflect other constraints, such as chemical constraints of colour producing pigments or exploitation of uncommon colors for a particular environment to increase advertisement [44].

The lack of a relationship between red reflectance (600-700nm) and VOC emission may result from the other benefits to a plant of investment in pigments that reflect at this range of the spectrum (e.g., anthocyanins). Advantages to red plant pigmentation include anti-fungicidal properties, photoprotection against UV damage, prevention of photoinhibition, and chromatic crypsis against dichromatic (red-green colour blind) herbivores [45,46,47]. Thus, while red pigmentation can be available to trichromatic animals as a cue of fruit ripeness, this may result from selective pressures other than disperser signalling.

Our finding that smaller fruits tend to invest heavily in VOC production may reflect the diminutive size of nocturnal, olfactory-driven mammals in Madagascar. Unlike most other tropical systems where the small end of the disperser size spectrum is dominated by avifauna, in Madagascar the smallest seed dispersing animals are mouse and dwarf lemurs of the family Cheirogaleidae [48]. Cheirogaleids are dichromatic (red-green colour blind) and nocturnal, and have been shown to rely heavily on olfaction during fruit selection and detection [19,20]. The fact that these animals are red-green colour blind may explain why red hues did not show a similar pattern to blue hues in our analysis.

Our results provide support for the idea that fruit traits may converge to simultaneously attract multiple dispersers with diverse sensory phenotypes by using both colour and odour signals when possible, or by decreasing olfactory signals when producing colours that all dispersers can see well. An important next step will be to record the behaviour of seed dispersers relative to fruit cues. In this forest there are five known seed dispersing mammals, three nocturnal and two cathemeral, [49,50] and four putative seed dispersing birds [51]. These seed dispersing mammals are cathemeral, nocturnal, and dichromatic, with highly developed olfactory apparatuses, and respond primarily to olfactory cues during fruit selection [20,52]. All frugivorous mammals in this system for which data are available on colour vision capabilities are dichromats, or red-green colour blind [52,53]. While dichromats are not able to distinguish between fruits in the red-green colour channel, they are able to distinguish fruits in the blue-yellow colour channel [54,55] and compelling evidence from studies of primate behaviour, genetics and ambient light measurements suggests that mammalian color vision is useful, even under nocturnal conditions [19,56,57,58]. Avian dispersers, on the other hand, are diurnal, tetrachromatic, visually oriented foragers, with a capacity to distinguish red from green, and into the UV spectrum [59]. Despite the fact that mammalian seed dispersing taxa in this forest are highly olfactorily-driven, [53], the colour that fruits produce at the expense of odour is one that is available to all seed dispersing animals in this system—blue.

## Supporting Information

S1 Fig The phylogeny that was adapted from APG III and other classifications (see references from text) and was used to test if there was s phylogenetic signal in the data we used in the subsequent analyzes. (PDF)

S1 File R code used in calculation of phylogeny. (TXT)

S2 File R code used in statistical analysis. (TXT)
