# Supplementary figures and images for: It’s Not Easy Being Blue: Are There Olfactory and Visual Trade-Offs in Plant Signalling?
Source: PLoS One. 2015 Jun 26;10(6):e0131725. doi: 10.1371/journal.pone.0131725 (PMC4482676; doi:10.1371/journal.pone.0131725)

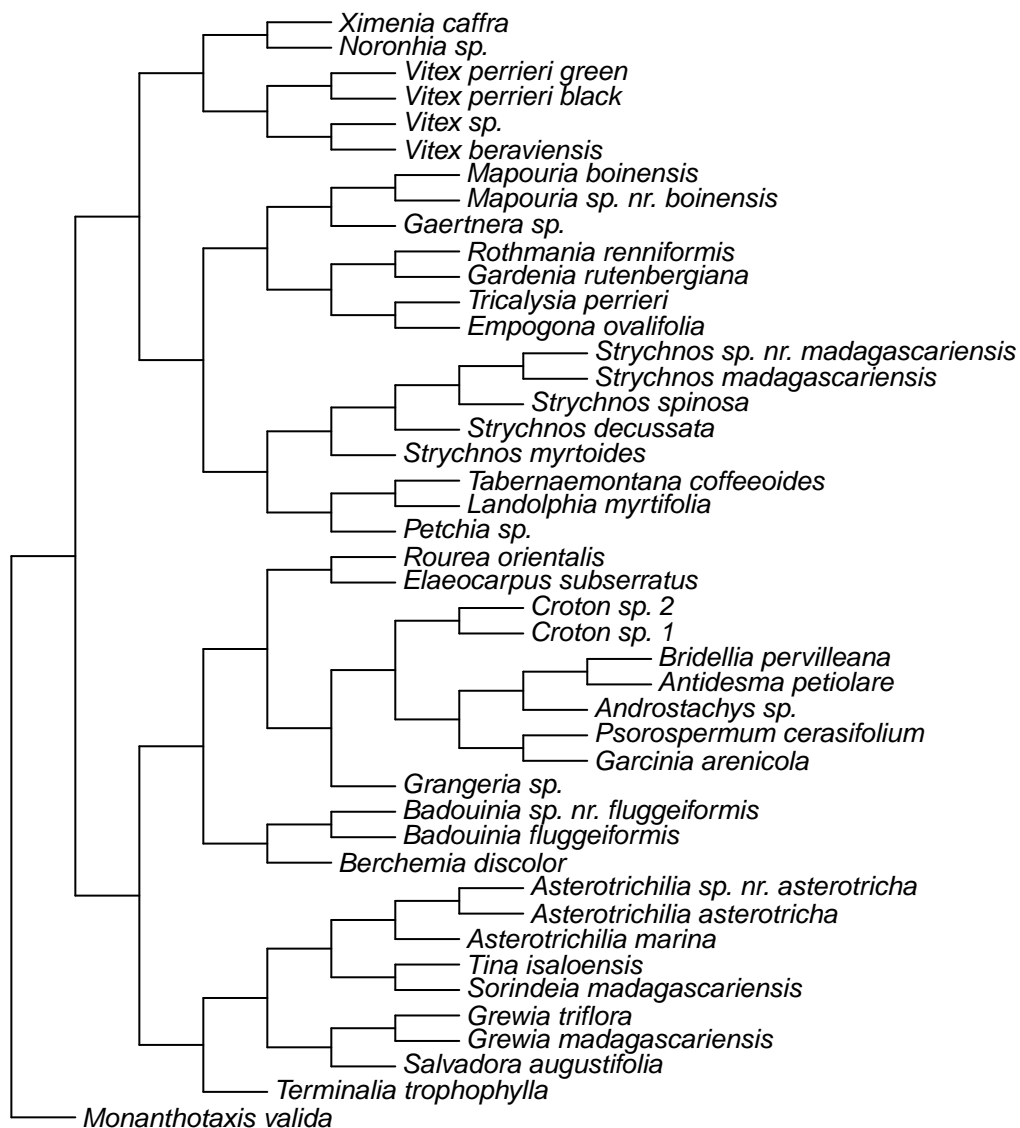

Supplement: S1 Fig — (PDF) [file pone.0131725.s001.pdf]
